# Factors Associated With Alzheimer's Dementia Diagnosis and Survival in Down Syndrome

**DOI:** 10.1111/jir.13230

**Published:** 2025-03-12

**Authors:** Olivia Pounds, Kate Theodore, Karen Dodd

**Affiliations:** ^1^ Trainee Clinical Psychologist at Royal Holloway University of London London UK; ^2^ Clinical Psychologist/Lecturer at Royal Holloway University of London London UK; ^3^ Surrey and Borders NHS Foundation Trust London UK

**Keywords:** Alzheimer's disease, dementia, Down syndrome, intellectual disability, risk factors, survival

## Abstract

**Background:**

People with Down syndrome (DS) have an increased risk for Alzheimer's disease (ad). Identifying factors associated with dementia onset and subsequent survival will support in understanding the disease profile, improving timely diagnosis, management, and care planning.

**Method:**

Variables associated with age at dementia onset and survival times were assessed in 279 adults with DS who accessed a community learning disability service. After outliers were removed, regression and hazard regression models were used for disease onset (*n* = 265) and survival times (*n* = 180), respectively.

**Results:**

Earlier age at first assessment and living with family predicted earlier age at diagnosis, which led to longer survival, post‐diagnosis. Epilepsy and living in a long‐stay hospital were associated with earlier mortality.

**Conclusion:**

Implications for clinical practice include reflections on the importance of early baseline assessments and caregiver awareness. Suggestions for future research include investigating intersectionality of social factors with genetics to better understand ad trajectories.

## 1 Introduction

People with Down syndrome (DS) are at a greater risk of Alzheimer's disease (ad), due to trisomy of chromosome 21 leading to the overproduction of amyloid beta (Doran et al. 2017; Wiseman et al. 2015). Typically, people with DS will show evidence of ad neuropathology by 40 years of age (Wisniewski et al. 1985), but not all will display the clinical symptoms associated with dementia (Ballard et al. 2016). Most commonly, the onset of dementia, presents around the age of 53–55 years (Antonarakis et al. 2020; Fortea et al. 2021). However, reports of onset in under 40s and in over 70s can occur (Ballard et al. 2016). This population can therefore provide unique insights into a longitudinal perspective of ad (Lott and Head 2019).

Progression occurs relatively quickly, such that within 5 years, almost all showing early signs of dementia progress to a dementia diagnosis, regardless of age (Iulita et al. 2022; Videla et al. 2022), with approximately 75% having a diagnosis of dementia by age 65 (Cipriani et al. 2018). Median survival time for people with DS, following dementia diagnosis, has been estimated at around 4 years (Sinai et al. 2018). Despite few known interventions to delay symptomology of dementia, early detection of dementia and understanding the various risk and protective factors can help with accessing relevant treatment and support, as well as planning for the future (The National Institute of Health Excellence 2018).

Akin to the general population, it is possible that the variation in clinical expression of dementia is linked to the additive and/or interactive effects of several risk factors (Zigman et al. 2008). In the DS population, suggested risk factors, as well as protective factors, remain inconclusive due to inconsistencies with findings (Sinai et al. 2018). For instance, a Cochrane review found that neither donepezil nor memantine proved more effective than placebo at reducing or delaying symptoms of ad in DS (Livingstone et al. 2015), whereas a more recent study found a significant survival advantage for those who had been prescribed either acetylcholinesterase inhibitors or memantine (Sinai et al. 2018). Similarly, sex differences in dementia have been controversial, with some finding females having an increased risk of dementia, possibly due to the drop in oestrogen levels during menopause, leading to earlier onset of dementia (Glasson et al. 2003; Schupf et al. 2006), particularly for those with menopause before 46 years old (Coppus et al. 2010). However, more recent studies contest findings that women are more at risk than men, with the possibility that perimenopausal symptoms are being misattributed to dementia (Devi 2018; Sinai et al. 2018; Takenoshita et al. 2023).

Other inconsistent findings include the risk of physical and psychiatric comorbidities (Altuna et al. 2021; Fonseca et al. 2014; Hithersay et al. 2019; McCarron et al. 2017; Menéndez 2005; Sinai et al. 2018) and lifestyle factors, which are often less explored compared to genetic and physical counterparts (Hamadelseed et al. 2022; Peven et al. 2022). Sinai et al. (2018) and Hithersay et al. (2019) found that people with DS, who were living with family, had significantly earlier ages at dementia diagnosis, which may be important to understand further, due to the high likelihood of relocation for people with DS, affecting consistency of care and care relationships (Patti et al. 2010).

### 1.1 Aims

Therefore, we aimed to analyse a large database of individuals with DS, who attended a specialist, community learning disability service for assessments of dementia, to better understand factors associated with dementia onset and survival time in this population.

### 1.2 Hypotheses

Based on age at dementia diagnosis presumably relating to, both, how quickly symptoms are picked up and the age of onset, it is hypothesised that less severe intellectual disability, living at home, having a family history of dementia and being female may predict earlier age at diagnosis. It is further hypothesised that protective factors for longevity of life will include being on anti‐dementia medication, having less severe intellectual disability, living at home and fewer accommodation moves, whereas risk factors will include mental and physical health comorbidities and an older age at diagnosis.

## 2 Methods

### 2.1 Sample and Demographics

Five hundred and seventy‐nine participants, who attended a community learning disability service both for specific dementia assessment or as part of other clinical assessments, which led to a dementia assessment, in the East and West Surrey catchment areas of England, between the years of 1993 and 2021, had data documented. The interest in dementia in people who have DS in Surrey was well known and regularly publicised to ensure that people with DS were referred for baseline assessment and re‐referred when there were any concerns. Data were entered on a database for pseudonymisation and longitudinal tracking of participants' assessments from first screening. For the current study, adults with DS, who also had a confirmed diagnosis of dementia at their most recent assessment (*n* = 265), were selected. For survival analyses, participants who had both a current diagnosis of dementia and had been assessed post‐diagnosis (*n* = 180) were included.

Table 1 displays demographics and clinical variables for the two samples (Table 1). There was considerable missing data (over 30%) for both groups in the variables of family history of dementia, ad medication and intellectual disability severity due to these data not always captured on assessment, or in the case of ad medication, due to a lack of development and use of medication during the earlier years of the study.

**TABLE 1:** Demographics and clinical variables for the diagnosed group, as well as the subset of the diagnosed group who were assessed post‐diagnosis.

	Diagnosed with dementia *N* = 265	Post‐diagnosis *N* = 180
Sex		
Female	107 (40.4%)	75 (41.7%)
Male	158 (59.6%)	105 (58.3%)
Died		
Yes	150 (56.6%)	144 (80%)
No	115 (43.4%)	36 (20%)
Level of intellectual disability		
Mild/borderline	23 (8.68%)	13 (7.2%)
Moderate	30 (11.32%)	16 (8.9%)
Severe	62 (23.3%)	41 (22.8%)
Profound	30 (11.3%)	45 (25%)
Missing	120 (45.2%)	65 (36.1%)
Residence at first assessment		
Family home	39 (14.7%)	25 (13.9%)
Supported living	36 (13.6%)	9 (5%)
Residential home	121 (45.7%)	96 (53.3%)
Specialist/nursing home	6 (2.3%)	3 (1.7%)
Hospital	26 (9.8%)	25 (13.9%)
Missing	37 (14.0%)	22 (12.2%)
Residence at diagnosis		
Family home	43 (16.2%)	28 (15.6%)
Supported living	40 (15.1%)	11 (6.1%)
Residential home	116 (43.8%)	90 (50%)
Specialist/nursing home	12 (4.5%)	8 (4.4%)
Hospital	34 (12.8%)	29 (16.1%)
Missing	20 (7.5%)	14 (7.8%)
Family history of dementia		
Yes	22 (8.3%)	11 (6.1%)
No	34 (12.8%)	16 (8.9%)
Maybe	8 (3%)	5 (2.8%)
Missing	201 (75.8%)	148 (82.2%)
ad medication		
Prescribed	71 (26.8%)	47 (26.1%)
Not prescribed	46 (17.4%)	25 (13.9%)
Missing	148 (55.8%)	108 (60%)
Number of physical health conditions (thyroid, epilepsy, chest, dysphagia, any sensory)		
0	42 (15.8%)	23 (12.8%)
1	76 (28.7%)	52 (28.9%)
2	73 (27.5%)	50 (27.8%)
3 or more	62 (23.4%)	47 (26.1%)
Missing	12 (4.5%)	8 (4.4%)
Epilepsy		
No	136 (51.3%)	86 (47.8%)
Early‐onset	59 (22.3%)	45 (25%)
Late‐onset	53 (20.0%)	38 (21.1%)
Missing	17 (6.4%)	11 (6.1%)
Sensory difficulties (either hearing, visual or both)		
Yes	158 (59.6%)	108 (60%)
No	55 (20.8%)	36 (20%)
Missing	52 (19.6%)	36 (20%)
Number of mental health diagnoses (anxiety, depression, schizophrenia, bipolar)		
0	147 (55.5%)	104 (57.8%)
1	48 (18.1%)	27 (15%)
2	15 (5.7%)	11 (6.1%)
3	1 (0.4%)	0 (0%)
Missing	54 (20.4%)	38 (22.1%)
Percentage of adults with a physical/mental health condition		
Anxiety	12.7%	11.7%
Depression	16.27%	15.79%
Schizophrenia	1.59%	0%
Bipolar	0.79%	0.58%
Sensory	62.7%	63.16%
Hypothyroid	37.3%	39.77%
Hyperthyroid	0.4%	0%
Pre‐existing epilepsy	36.51%	40.35%
Chest infections	17.86%	21.05%
Dysphagia	12.7%	14.04%
Number of accommodation moves		
0	94 (35.5%)	73 (40.6%)
1	76 (0.7%)	49 (27.2%)
2	32 (12.1%)	23 (12.8%)
3	27 (10.2%)	16 (8.9%)
4+	10 (3.8%)	5 (2.8%)
Missing	26 (9.8%)	14 (7.8%)
Mean (*SD; range*) age at first assessment	49.44 (*8.38; 48*)	50.46 (*7.83;41*)
Mean (*SD; range*) age at dementia diagnosis	55.25 (6.83; *37*)	55.58 (6.57; *32*)
Mean (*SD; range*) age at death	60.27 (*6.49; 39)*	60.52 (*6.13; 28*)
Mean (*SD; range*) number of assessments	5 (*3.36; 15*)	5.6 (*3.55; 15*)

### 2.2 Ethics Review and Approval

The study was approved by the Health Research Authority on 5 May 2022 with the registration number 22/HRA/1050. Ethical approval for the longitudinal study was granted by the Mid‐Surrey Health Authority in 1993 for the collection of data included in this paper, which allowed us to collect pseudonymised retrospective data without requiring individual consent.

### 2.3 Variables of Interest

Variables extracted from medical records and clinical assessment were date of birth, sex (m/f), family history of dementia (y/n), date of death or date of last assessment if the participant was alive, mental and physical health comorbidities (epilepsy, thyroid disease, respiratory disease, dysphagia, hearing loss, visual loss, anxiety, depression, schizophrenia and/or bipolar disorder), accommodation type (family home, supported living, residential home, nursing home, long‐stay hospital), number of residential moves between all accommodation types, prescription of anti‐dementia medication (y/n) and severity of intellectual disability (borderline/mild, moderate, severe or profound/unable to be tested). Severity of intellectual disability was calculated from scores on the British Picture Vocabulary Scale (BPVS; Dunn et al. 1982) using the equation in Ezard et al.'s (2022) paper and DSM‐IV criteria (APA 2000). Further information on the variables included in analyses can be found in Table S1.

### 2.4 Diagnosis of Dementia

A dementia diagnosis was made by the assessing clinical team based on the International Classification of Diseases (ICD) criteria (World Health Organisation 1993), supported by a detailed history of cognitive and functional changes, medical history, psychiatric evaluation and neuropsychological testing. The Neuropsychological Assessment of Dementia in Adults with Intellectual Disabilities (NAID; Crayton et al. 1998) was used as a validated battery of simple tests covering memory, orientation, language and praxis (Oliver et al. 2022), and part of the Cambridge Cognitive Examination adapted for individuals with Down syndrome (CAMCOG‐DS; Ball et al. 2004) was used when participants were more cognitively able and had scored at the ceiling of the NAID. Adaptive behaviour was captured by an informant who knew the participant for at least 6 months using the Hampshire Assessment for Living with Others (HALO; Shackleton 1987). This measure has behavioural descriptors for every scoring element, which gives better reliability and validity than other comparable measures (Kelly et al. 2006).

A clinical judgement was made by the team as to whether the person with DS had a likely dementia diagnosis at the point of assessment, in accordance with published best‐practice guidelines at the time. The most up‐to‐date version of guidance has been published by the British Psychological Society (BPS) and the Royal College of Psychiatrists (RCP) (2015). At each assessment, a record of whether someone had no dementia, suspected dementia or a dementia diagnosis was recorded. Date of dementia diagnosis was recorded as the date from which a consistent dementia diagnosis was given at all subsequent assessments. Changes from a dementia diagnosis to a suspected diagnosis (*n* = 4) or no dementia (*n* = 2) were rare. It was more common for suspected dementia to be recategorised as no dementia at a later assessment (*n* = 24).

### 2.5 Analysis

*T*‐tests, Pearson's correlation or ANOVA was used to test the association between participant demographics and clinical variables and age at dementia diagnosis. Significant variables were dummy coded and entered at an alpha level of 0.05 into a regression analysis to control for confounding. Games–Howell post hoc tests were chosen when there were unequal variances. Where unequal sample sizes were present, Tukey post hoc tests were used to assess for differences in groups (Field 2013).

To estimate median survival the Kaplan–Meier (log‐rank) method was used. Univariate Cox regression models were fitted for each predictor for survival time after diagnosis. Significant predictors were then entered in a stepwise approach into a multivariate model to control for confounding. For the proportional hazard assumption, deviations from a linear trend were taken to indicate a time‐dependent covariate, which were then adjusted for (Allison 2010).

A priori power analyses for regression models, Cox regression and *F‐*tests showed sample sizes were sufficient. All analyses were carried out using SPSS v25.

## 3 Results

### 3.1 Predictors of Age at Diagnosis and Survival

#### 3.1.1 Outliers

**FIGURE 1:**
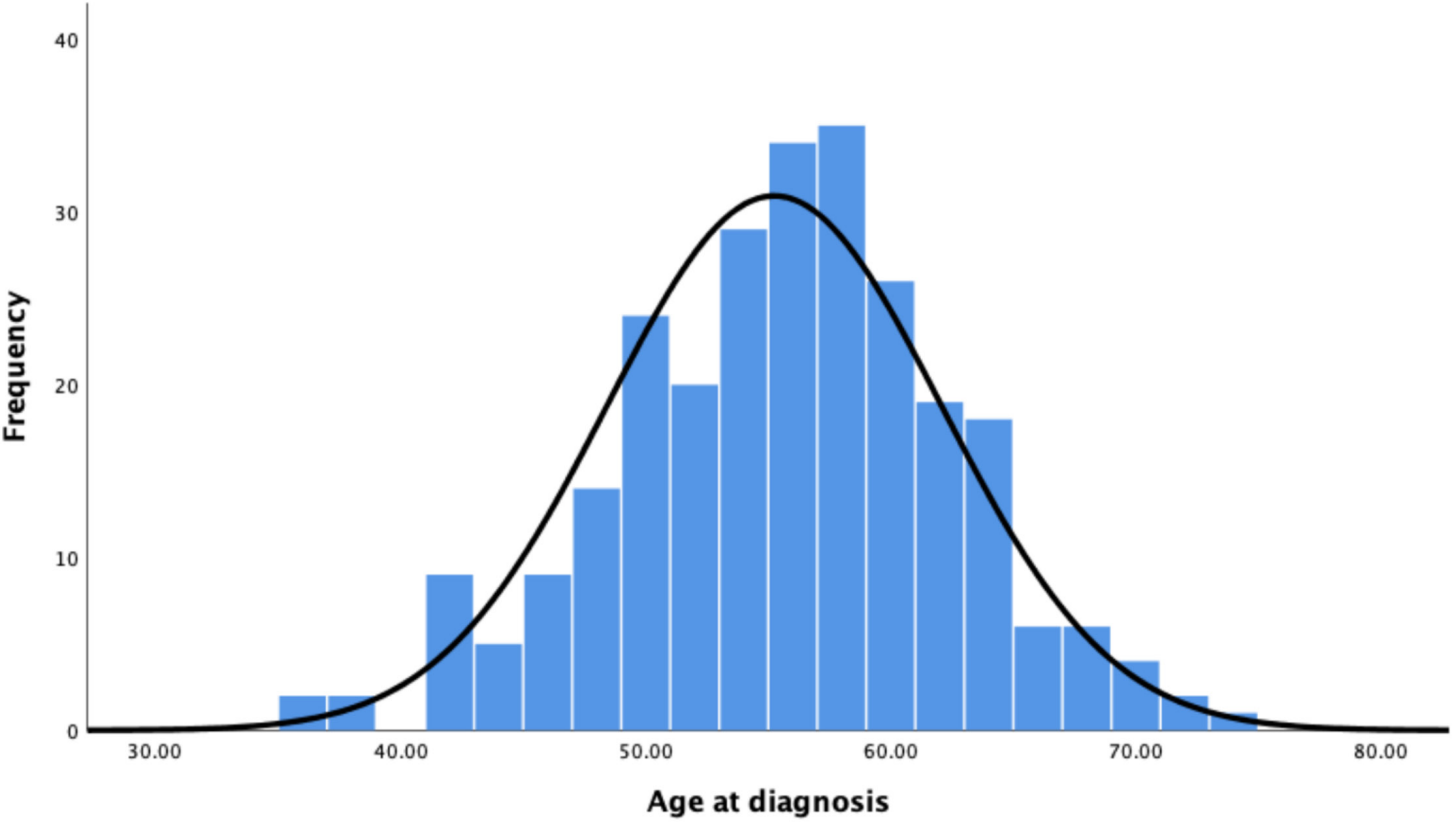
Distribution of ages at dementia diagnosis (*n* = 265).

From the sample of 279 with a dementia diagnosis, five people had missing ages at diagnosis and were removed. Outliers were identified on the extreme ends, which reflected possible late diagnosis due to late age at first assessment or perhaps too early a dementia diagnosis that may have reflected a differential diagnosis. At the higher end, two extreme cases were identified, both over 75 years old and both with an age at first assessment over 70 years old. At the lower end, only those younger than 35, at dementia diagnosis, were identified as extreme cases, and thus, those above 35 were included due to the neuropathology of AD often already present in those who are 40 years old (Wisniewski et al. 1985). After removing extreme outliers (greater than 75; *n* = 2) and (less than 35; *n* = 7) assumptions of normality were met. Mean age at dementia diagnosis was 55.25 (*SD* = 6.83) with an IQR between 51 and 59 (Figure 1). Outliers were therefore removed for subsequent analyses in order to meet the requirement of normally distributed data for statistical analyses.

#### 3.1.2 Predictors of Age at Diagnosis

Significant differences for age at diagnosis were found amongst the variables: residence at first assessment (*F*_4,223_ = 4.53, *p* = 0.002, η_p_^2^ = 0.8), residence at diagnosis (*F*_4,240_ = 3.9, *p* = 0.004, η_p_^2^ = 0.6), and level of intellectual disability severity (*F*_3,167_ = 3.32, *p* = 0.02, η_p_^2^ = 0.6). Age at first assessment was also found to be strongly positively correlated to age at diagnosis *r* = 0.69, *p* < 0.001.

Post hoc tests revealed those with a profound intellectual disability had a higher age at diagnosis (*M* = 57.18, *SD* = 7.2) as compared to those with moderate intellectual disability (*M* = 52.57, *SD* = 7.27, *p* < 0.05), but not compared to those with mild or severe intellectual disability, although this may have been due to loss of statistical power (Table 2). Other significant findings that were associated with an earlier age at diagnosis were living with family at the first assessment (*M* = 51.82, *SD* = 6.34), as compared to living in a residential home (*M* = 56.93, *SD* = 6.58, *p* < 0.001), and living with family at the point of diagnosis (*M* = 52.77, *SD* = 6.51), as compared to living in hospital (*M* = 57.41, *SD* = 7.36, *p* < 0.05) or a residential home (*M* = 56.41, *SD* = 6.43, *p* < 0.05). Those with missing scores across all variables did not significantly differ from the sample reported on (Table 2).

**TABLE 2:** Differences in age at diagnosis for reported variables.

	*n*	Age at diagnosis M (SD)
Sex		
Female	107	54.88 (6.28)
Male	158	55.45 (7.25)
Level of intellectual disability		
Mild/borderline	23	53.91 (6.93)
Moderate	30	52.57 (7.27)*
Severe	62	55.03 (5.9)
Profound/unable to test	56	57.18 (7.2)*
Missing	94	55.31 (6.91)
Family history of dementia		
Yes	22	54.59 (5.61)
No	34	53.74 (8.27)
Maybe	8	56.38 (6.44)
Missing	201	55.49 (6.87)
Residence at first assessment		
Family home	39	51.82 (6.34)***
Supported living	33	54.18 (6.59)
Residential home	124	56.93 (6.58)***
Nursing home	6	54.5 (7.71)
Hospital	26	55.96 (6.65)
Missing	37	53.59 (7.15)
Residence at diagnosis		
Family home	43	52.77 (6.51)*
Supported living	40	53.98 (6.37)*
Residential home	116	56.47 (6.43)*
Nursing home	12	53.42 (7.51)
Hospital	34	57.41 (7.36)*
Missing	20	53.1 (7.71)
Number of moves		
0	94	55.84 (6.14)
1	76	55.32 (6.61)
2	32	56.68 (5.97)
3	27	54.85 (8.01)
4+	10	56.8 (6.68)

* *p* < 0.05.

** *p* < 0.01.

*** *p* < 0.001.

When controlling for multiple variables, age at first assessment explained the most variance in the model (*F*_1,157_ = 168.76, *p* < 0.001 *adjusted R* ^*2*^ = 0.52), with a younger age at first assessment predicting a younger age at diagnosis (β = 0.69, *p* < 0.001). Only residence at diagnosis explained a further significant increase in variance (*F*_1,155_ = 7.72, *R* ^*2*^ change = 0.02, *p* = 0.02). Living in the family home at diagnosis predicted younger age at diagnosis (β = −0.17, *p* = 0.002), as did living in a nursing home (β = −0.13, *p* = 0.02), compared to other locations.

#### 3.1.3 Predictors of Survival

Just under 80% of people with a dementia diagnosis had died, with a mean survival of 4.81 years (95% CI [4.22, 5.4]) and median survival of 4 years following diagnosis (IQR: 2–7).

**FIGURE 2:**
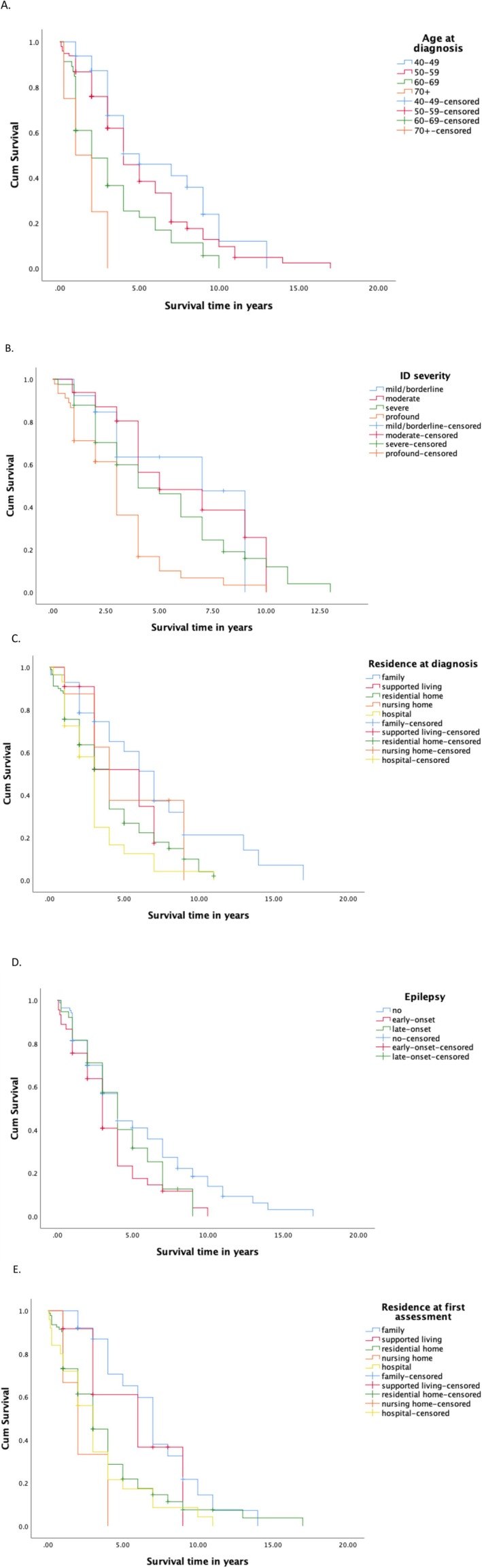
Survival Kaplan–Meier curves for the five significant predictors of survival time with censoring indicated by the crosses in the lines. (A) Age at diagnosis. (B) Intellectual disability (ID) severity. (C) Residency at diagnosis. (D) Epilepsy type. (E) Residency at first assessment.

Survival analysis using Kaplan–Meier estimates showed significantly greater survival times, post‐diagnosis, for 40–49 year olds (K‐M median = 5, IQR: 3–9) compared to over 70s (K‐M median = 1, IQR: 0.25–0.2; *p* < 0.01), for those without epilepsy (K‐M median = 4, IQR: 2–8), compared to those with epilepsy (K‐M median = 3, IQR: 2–5; *p* < 0.05), and for milder intellectual disability severity (K‐M median = 7, IQR: 3–9), compared to profound intellectual disability (K‐M median = 3, IQR: 1–4; *p* < 0.01). Those living at home at their first assessment had greater survival times (K‐M median = 7, IQR: 4–9) compared to those in long‐stay hospitals (K‐M median = 3, IQR: 1–4; *p* < 0.01) and nursing homes (K‐M median = 2, IQR: 1–4; *p <* 0.01*)*. Those living at home at diagnosis (K‐M median = 7, IQR: 3–9) also survived longer than those living in long‐stay hospitals (K‐M median = 3, IQR: 1–3; *p* < 0.01) (Figure 2). Although no other significant effects were found on survival, males had lower mean survival times than females, taking ad medication had a higher mean survival time compared to not taking medication, and not having a physical health comorbidity was associated with higher mean survival time. Significant variables (epilepsy type, residency at diagnosis, residency at first assessment and ID severity) were then entered into univariate Cox proportional hazard models, controlling for age at diagnosis, and adjusted for time dependence, if needed. Hazard ratios revealed only epilepsy and residency at diagnosis remained significant. Multivariate Cox proportional hazards regression was then run to include only those variables significantly associated with survival (Table 3). Age at diagnosis remained significant, predicting the most variance in the model, with later age at diagnosis predicting a greater hazard for death (χ2(9) = 28.65, *p* = 0.001). Epilepsy and residency at diagnosis, entered together, found a significant improvement to model fit, above that of age at diagnosis (χ2 change(6) = 15.42, *p* = 0.02). Further scrutiny revealed both early and late onset epilepsy were significant for early death (*HR* = 1.06, *p* = 0.02; *HR* = 1.05, *p* = 0.01), respectively. Living in hospital at the time of diagnosis also predicted a significantly greater hazard for death compared to living with the family (*HR* = 1.98, *p* = 0.04).

**TABLE 3:** Hazard ratios for survival after dementia diagnosis; controlling for covariates and confounds (*n* = 134 events).

	Hazard ratio [95% CI]	*p*
Age at diagnosis		
40–49	Reference	
50–59	1.36 [0.81, 2.29]	> 0.05
60–69	2.22 [1.22, 4.04]	0.01
70+	3.58 [1.16, 11.08]	0.03
Epilepsy (*time‐varying*)		
No	Reference	
Early‐onset	1.06 [1.01, 1.12]	0.02
Late‐onset	1.05 [1.01, 1.08]	0.01
Residence at diagnosis		
Family	Reference	
Supported	1.07 [0.42, 2.77]	> 0.05
Residential	1.41 [0.82, 2.4]	> 0.05
Nursing	0.77 [0.30, 1.98]	> 0.05
Hospital	1.98 [1.04, 3.74]	0.04

## 4 Discussion

This study analysed a large sample of people with DS living in Surrey, collected between 1993 and 2021, for predictors of age at dementia diagnosis and subsequent survival. It is part of one of the largest to follow people with DS over three decades, offering important insights into ageing with DS.

### 4.1 Age at Diagnosis

Similarly to Sinai et al. (2018), Videla et al. (2022) and Iulita et al. (2022), average age at dementia diagnosis was in the mid‐50s. However, in keeping with these papers, variability (36–73) was also broad. Age at diagnosis was associated with the age at which someone was assessed, such that an earlier diagnosis was more likely if the person had an earlier baseline assessment. This supports guidance that first assessment should occur around the age of 30–35, since any earlier, and development may still be occurring (The British Psychological Society and the Royal College of Psychiatrists 2015; Carr 2000). However, the average age at first assessment, in this study, was found to be around 49, suggesting that there is a delay in people being seen within the recommended guideline times. Perhaps the data are biased by inclusion of a sample from earlier time periods that predate current good practice guidance (BPS/RCP 2015).

Living in a nursing home and family home were also predictors of an earlier age at diagnosis. This finding supports our hypothesis that those living at home may have more consistency with carers, who are able to better report on changes occurring (BPS/RCP 2015; Hithersay et al. 2019). For those in nursing homes, the group size was very small, which may indicate an incidental finding.

Although not significant after controlling for other variables, having moderate intellectual disability was also a predictor of a younger age at diagnosis, which has been observed by others (McCarron et al. 2017). In keeping with the brain reserve hypothesis (Katzman et al. 1988), it is possible that milder intellectual disability severity may be protective and therefore delay dementia. This trend does not continue for more severe forms of intellectual disability due to the challenge in detecting declines on scales. Indeed, those individuals with more severe and profound intellectual disability had lower scores across measures, suggesting greater challenges for clinicians in predicting reliable change from baseline. Carers may also be less likely to notice changes for people with more severe intellectual disability. Indeed, the average age at first assessment was seen to be later with worsening intellectual disability severity.

Little in the way of other factors, included in this study, appeared to influence age at diagnosis.

### 4.2 Survival Post‐Diagnosis

Median survival time, after a dementia diagnosis, was 4 years, which is consistent with others who have found mean disease duration to be from 3.2 to 4.6 years (Iulita et al. 2022). After adjusting for the effects of other predictors, only age at diagnosis, epilepsy status and residence at first assessment remained significant. Expectedly, as individuals who are older are likely to have a naturally lower survival time, later age at diagnosis meant less survival time, particularly for those over 60. The sample also included four individuals over 70, which is rarer for studies in DS, and revealed an accelerated decline in longevity with age, with a median survival of just 1 year for those in their seventh decade. These estimates can support discussions around prognosis and facilitate preparations for future care with people who have DS and their families/carers (Haaksma et al. 2020; Lemoine and Schneider 2022).

Being in a long‐stay hospital significantly predicted shorter life expectancy, as compared to living with family. It is likely that these individuals were born in an earlier era, when life expectancy was lower, as all long‐stay hospitals in England have since been closed. Living in a long‐stay hospital and being institutionalised, compared to living with family, may also be associated with poorer physical and mental health due to potentially less stimulation and neglect leading to lower life expectancy (Houck and Dracobly 2022; Hubert and Hollins 2010).

The finding that epilepsy predicted shorter life expectancy is in line with previous research that suggests comorbid epilepsy and dementia are associated with a greater risk of mortality (McCarron et al. 2017; Sinai et al. 2018). Interestingly, we found that early‐onset epilepsy, which is not associated with ad, was also predictive of earlier death, which was not found by others (Sinai et al. 2018). Although speculative, medications used to treat epilepsy, such as phenytoin, may have negatively impacted on lifespan due to the potential for side effects (Altuna et al. 2021; Herranz et al. 1988). Additional research into this area is required.

### 4.3 Strengths and Limitations

The main strength of the study is the representativeness of the sample of individuals with DS living in the Surrey area, who presented with a range of intellectual disability severity, residential locations and comorbidities. The study also captured data over a long follow‐up period, revealing trends for some people with DS into their 70s. However, data on ethnicity were not routinely collected, which may affect the representativeness of our findings and limit the generalisability to the broader population, within the United Kingdom currently, or to international populations. Given that ethnicity might interact with genetic disposition and health inequality in people with DS, this requires further exploration.

There are several other limitations to this study. Firstly, it was not possible to control for APOE genotype, which has been shown as a significant predictor of both age at dementia diagnosis and mortality in DS (Prasher et al. 2008). There was an attempt to control for family history of dementia and to mitigate this effect; however, data were limited. This study also found little to suggest that anti‐dementia medication was associated with significantly longer disease duration; however, there were considerable missing data due to these medications not having been developed until later periods in the study.

Secondly, the method for predicting intellectual disability severity followed a recently suggested approach that uses IQ cut‐offs (Ezard et al. 2022). However, the DSM‐V suggests that these IQ cut‐offs are arbitrary and that severity of intellectual disability should include consideration of wider adaptive functioning (American Psychiatric Association 2000; Greenspan and Woods 2014). However, we also compared intellectual disability severity ranges to another method that uses age‐associated norms (Oliver et al. 2022), and bar a few exceptions, results were generally consistent.

### 4.4 Implications for Clinical Practice and Future Research

This study reveals different practice implications. Firstly, there was a wide variance in the age at which dementia diagnoses were made. This was partly due to the age at which someone was first assessed, supporting guidelines in terms of establishing an early baseline assessment for all individuals with DS (BPS/RCP 2015). Given that some individuals were in their 30s when diagnosed, this suggests such assessments should be around 30–35 years old. However, the average age of diagnosis was around 55 years old, providing a 15‐year window for potential pharmaceutical intervention from when neuropathology is expected to be present (Wisniewski et al. 1985). The data also show that diagnoses are being made for some people in their 60s or 70s. It was not possible to determine whether this was due to these individuals developing dementia later in life, whether diagnoses were delayed or whether they had recently moved into the area. This is relevant in terms of improving effective care for people with DS, as this study hypothesises that an earlier dementia diagnosis will lead to a greater window for improved dementia care, as duration of disease will be extended. As seen in this study, those diagnosed in their 60s and 70s had just 1–2 years of survival.

Secondly, given that living with family also led to an earlier dementia diagnosis, above that of age at first assessment, this suggests that closer and more consistent carer–client relationships can potentially lead to more efficient care and better informant reporting. To improve this in residential services, carer training and the use of dementia checklists at annual reviews are recommended (BPS/RCP 2015).

Finally, some research suggests that there are different dementia trajectories in DS depending on sex (Coppus et al. 2010). However, the current study did not find strong sex effects on diagnosis or longevity post‐diagnosis. There is a need to look at perimenopausal women with DS to see whether signs of menopause are being misattributed to dementia.

## 5 Conclusions

Overall, this study provides evidence to suggest that the age at which a dementia diagnosis is made is strongly associated with the age at first assessment. Living with family also seemed to infer an advantage, irrespective of health comorbidities or intellectual disability severity. Consistent carers, and perhaps other social factors related to living with family, can lead to improved diagnosis time, which leads to better longevity post‐diagnosis. This then allows for better care planning and a wider window for support.

Future studies may wish to focus on the intersectionality of genetic and health risk factors with social factors, including type of residence, which may be frequently neglected from studies. As shown in this study, including social factors can ensure better understanding of the risk and protective factors associated with mortality in DS, via relatively amenable changes, such as supporting closer carer–client relationships and providing education around the importance of early assessment. Strengthening this research will help support with best practice guidance for people with DS, leading to improved outcomes (National Institute of Health Excellence 2018).

## Conflicts of Interest

The authors declare no conflicts of interest.

## Supporting information


**Table S1** Included variables and their corresponding definitions.

## Data Availability

The data that support the findings of this study are available from the corresponding author upon reasonable request.
