# Supplementary material for: Factors Associated With Alzheimer's Dementia Diagnosis and Survival in Down Syndrome
Source: J Intellect Disabil Res. 2025 Mar 12;69(6):489–501. doi: 10.1111/jir.13230 (PMC12051232; doi:10.1111/jir.13230)
Supplement: Supplementary file 1 — Table S1 Included variables and their corresponding definitions. [file JIR-69-489-s001.docx]

Supplementary document

Table: Included variables and their corresponding definitions

| **Included variable** | **Definition** | |
| --- | --- | --- |
| Sex | Male or female at birth | |
| Age at baseline | Age at first assessment with the service | |
| Total assessments | Total number of recorded assessments | |
| Age at death | Age recorded at death | |
| Predicted age at present | Estimated current age, measured from date of birth | |
| Age at diagnosis | Age at which individuals were diagnosed with dementia | |
| Survival time | Calculated time from diagnosis to death, or until the last assessment, if censored. Censored in survival analysis denotes those who are still alive at the end of the data collection period | |
| Total accommodation moves | Total number of accommodation moves as recorded at first assessment | |
| Family history | Whether they had a known family member with dementia | |
| AD medication | Whether they had been prescribed anti-dementia medication at any time | |
| Sensory difficulties | Whether they had any hearing or visual impairments as recorded at last entry | |
| Epilepsy type | If they had epilepsy, either pre-dementia and therefore ‘early-onset’, or after diagnosis and therefore ‘late-onset’, as recorded at last entry | |
| Physical health conditions | The number of physical health conditions at last assessment, from the list of hypothyroidism, hyperthyroidism, epilepsy, chest, dysphagia, hearing loss and/or visual loss | |
| Mental health diagnoses | The number of mental health conditions at last assessment from the list of anxiety, depression, schizophrenia and/or bipolar disorder | |
| Residency at first assessment | The location of residence at their first assessment from the following list:  *Family home*: living with family/relatives; *Supported living*: living in supported accommodation; *Residential home*: living in a residential home; *Nursing home*: living in a nursing home; *Hospital*: living in a long-stay learning disability hospital, as per policy surrounding the living arrangements for people with learning disabilities at the time | |
| Residency at first diagnosis | The location of residence at dementia diagnosis from the following list:  *Family home*: living with family/relatives; *Supported living*: living in supported accommodation; *Residential home*: living in a residential home; *Nursing home*: living in a nursing home; *Hospital*: living in a long-stay learning disability hospital, as per policy surrounding the living arrangements for people with learning disabilities at the time | |
| Level of intellectual disability | The estimated level of intellectual disability as calculated from the highest recorded score on the British Picture Vocabulary Scale (BPVS) with scores placed into the following categories from the DSM-IV:  *Borderline/mild*: >50  *Moderate*: 35-50  *Severe*: 20-35  *Profound/unable to be tested*: <20 | |
|  | |  |
